# Clinical and imaging approach to headache in patients with multiple sclerosis

**DOI:** 10.1186/1129-2377-14-S1-P106

**Published:** 2013-02-21

**Authors:** C Voiticovschi-Iosob, I Moldovanu

**Affiliations:** 1 State Medical and Pharmaceutical University "Nicolae Testemitanu", Moldova, Republic of

## Background and aims

Headache has a high frequency in patients (P) with multiple sclerosis (MS), but its underlying mechanism is still unclear. The study aimed to diagnose headache types in P with MS and ascertaining a correlation with neuroimaging changes in these P.

## Materials and methods

38 P diagnosed with MS were screened for headache. Headache diagnosis was established in compliance with the International Headache Society diagnostic criteria ed. II 2004. Pain intensity was subjectively assessed by patients on a visual analogical scale (1-10 points). Patient disability assessment was conducted according to the EDSS scale. All P were investigated with brain MRI (1,5T).

## Results

Of the 38 P: 31 (81,57%) had headache (3 men and 28 women, average age 30,5 and 29,7 years respectively). P were repartizated in 3 groups: group I- 18 P (58,06%) with MS and tension-type headache, group II- 9 P (29,03%) with MS and migraine, group III- 4 P (12,90%) with MS and mixed headache: tension-type and migraine. The brain MRI 1,5T detected supratentorial demyelinating lesions located in the frontal lobes and white matter of all 9 P with migraine. Foci of demyelination in the brainstem, red nucleus, substantia nigra and periaqueductal gray matter were found in 8 P (88,88%) with migraine. And only 4 of P (22,22%) with tension-type headache had demyelinating lesions in the brainstem. There wasn’t found an association between EDSS value and headache severity.

## Conclusion

The results highlight the prevalence of primary headache- 81,57% in P with MS. There has been noted a significant correlation between headache severity and the presence of demyelinating lesions in brainstem, substantia nigra, red nucleus and periaqueductal gray. These lesions were more common in P with migraine and this fact could explain its comorbidity in these P.
